# Comparative Metabolomics Analysis of Cervicitis in Human Patients and a Phenol Mucilage-Induced Rat Model Using Liquid Chromatography Tandem Mass Spectrometry

**DOI:** 10.3389/fphar.2018.00282

**Published:** 2018-04-04

**Authors:** Xiaoyong Zhang, Junmao Li, Bin Xie, Bei Wu, Shuangxia Lei, Yun Yao, Mingzhen He, Hui Ouyang, Yulin Feng, Wen Xu, Shilin Yang

**Affiliations:** ^1^Jiangxi University of Traditional Chinese Medicine, Nanchang, China; ^2^State Key Laboratory of Innovative Drug and Efficient Energy-Saving Pharmaceutical Equipment, Nanchang, China; ^3^Nanchang Institute for Food and Drug Control, Nanchang, China; ^4^Second College of Clinical Medicine, Guangzhou University of Chinese Medicine, Guangzhou, China

**Keywords:** cervicitis, UPLC-QTOF-MS/MS, metabolomics, metabolic pathway, multivariate analysis

## Abstract

Cervicitis is an exceedingly common gynecological disorder that puts women at high risk of sexually transmitted infections and induces a series of reproductive system diseases. This condition also has a significant impact on quality of life and is commonly misdiagnosed in clinical practice due to its complicated pathogenesis. In the present study, we performed non-targeted plasma metabolomics analysis of cervicitis in both plasma samples obtained from human patients and plasma samples from a phenol mucilage induced rat model of cervicitis, using ultra-performance liquid chromatography coupled to quadrupole time-of-flight tandem mass spectrometry. In addition to differences in histopathology, we identified differences in the metabolic profile between the cervicitis and control groups using unsupervised principal component analysis and orthogonal projections to latent structures discriminant analysis. These results demonstrated changes in plasma metabolites, with 27 and 22 potential endogenous markers identified in rat and human samples, respectively. The metabolic pathway analysis showed that linoleic acid, arachidonic acid, ether lipid, and glycerophospholipid metabolism are key metabolic pathways involved in cervicitis. This study showed the rat model was successfully created and applied to understand the pathogenesis of cervicitis.

## Introduction

Cervicitis is an extremely common gynecological disorder in women aged 20–40 years, which puts women at high risk of sexually transmitted infections and can induce a series of reproductive system disorders, such as endometritis, salpingitis, pelvic inflammatory disease, chorioamnionitis, and other complications during pregnancy (Jayakumar, 2015). In clinical practice, cervicitis is divided into acute and chronic cervicitis and the latter comprises the majority of cases. Chronic cervicitis has been reported to be associated with various steps in the progression of cervical cancer, including cellular transformation, and promotion of survival, proliferation, invasion, angiogenesis, and metastasis (Schmauz et al., 1989; Castle et al., 2001; Aggarwal et al., 2006). Currently, the causes of cervicitis are varied and still uncertain for most patients. According to the Sexually Transmitted Diseases Treatment Guidelines published by the Centers for Disease Control and Prevention (CDC) of the United States (Workowski and Bolan, 2015), cervicitis is always attributed to chlamydia trachomatis, *M. genitalium* and *Neisseria gonorrheae* infections, merging with trichomonas vaginalis and genital herpes simplex virus. *M. genitalium* infection is significantly associated with increased risk of cervicitis and can potentially cause reproductive complications (Lis et al., 2015). Persistent abnormal vaginal flora, vaginal lavage, or other types of chemical irritants may also induce the development of cervicitis in some cases. The symptoms of cervicitis, which are initially diagnosed during pelvic examination, consist of the appearance of mucopurulent cervical discharge and/or easily induced bleeding on sample collection.

In recent years, the diagnosis of cervicitis is commonly inaccurate, and women with the disorder may not receive effective treatment. Metabolomics has attracted increasing attention among researchers and become an effective and accurate tool for identifying biomarkers of many diseases, including cancers, metabolic disorders, and infectious diseases (Huang et al., 2013; Cui et al., 2016; Shao et al., 2016; Liu et al., 2017; Zhou et al., 2017). Unlike traditional biochemical methods tending to concentrate on single metabolites, metabolomics reveals a collection of small molecules such as lipids, amino acids, and organic acids, which provides comprehensive information on pathophysiological processes, pharmacological intervention, and metabolic kinetics. As for cervicitis, few metabolomics studies have revealed the pathogenesis and its relevant diagnostic biomarkers. Previous study found serum metabolites contributing to discriminations between cervical cancer, cervical intraepithelial neoplasia, and chronic cervicitis (Ye et al., 2015). There is an investigation showed biomarkers for cervical cancer diagnosis and screening based on metabolomics and transcriptomics (Yang et al., 2017). However, what is the pathogenesis of cervicitis and what is the diagnostic biomarker between health and cervicitis patients? It remains unknown and it would intervene the research of cervicitis in clinical. The use of biomarkers in the diagnosis of cervicitis can effectively reduce the probability of oncogenesis and development of cervical cancer. Therefore, a comprehensive understanding of cervicitis is urgently needed to identify the representative biomarker for accurate diagnosis.

In general, metabolomics studies depend on various high-throughput techniques, such as liquid chromatography tandem mass spectrometry (LC-MS/MS), nuclear magnetic resonance spectroscopy (NMR), and gas chromatography-mass spectrometry (GC-MS) (Lee et al., 2016; Lindahl et al., 2017; Reisetter et al., 2017). An ultra-performance liquid chromatography quadrupole time-of-flight mass spectrometry (UPLC-QTOF-MS/MS) technology platform has been widely used in metabolomics for its high sensitivity and selectivity, better peak resolution, low ion suppression and high-throughput, which is well-suited for large-scale untargeted metabolomics studies (Lu et al., 2008). Previous studies has revealed UPLC-QTOF-MS/MS with high-resolution could provide massive accurate chemical information for thousands of compounds which greatly benefit metabolomics that aim to discover biomarkers and their pathways (Yin and Xu, 2014; Fu et al., 2017).

In fact, to investigate mechanism of action of the drugs or pro-drugs in clinical, it could be time-consuming, laborious, and difficult to recruit patients. Researchers often apply animal models to study related diseases in the early stage. A rat model could be easier and more repeatable to operate with relevant experiments. Some tests are not ethical to perform on human and therefore a rat model is good. To compare similarities between human patients and a rat model may be indicative for capturing the metabolic pathway in organism and specific biomarkers. Based on the similarities, a further clinical research could be more ambitious and targeted. To investigate the detailed metabolomics profile of cervicitis and identify potential biomarkers, a plasma metabolomics was applied using UPLC-QTOF-MS/MS with high resolution. In our study, we compared the metabolomics profiles of cervicitis in both human patients and a rat model of cervicitis induced by phenol mucilage.

## Materials and methods

### Materials

Methanol and acetonitrile (analytical gradient grade) for lipid chromatography were purchased from Merck (Darmstadt, Germany). Deionized water was produced using a Mill-Q ultrapure water system (Millipore, USA). HPLC grade formic acid was obtained from Tianjin Kermel Chemical Reagent Company (Tianjin, China). 2-Chloro-L-phenylalanine for use as an internal standard (IS) was provided by Shanghai Macklin Biochemical Company (Shanghai, China). Standard eicosapentaenoic acid, palmitoleic acid, glycocholic acid, gamma-Linolenic acid, arachidonic acid, thymidine, linoleic acid, L-Phenylalanine, L-Tryptophan, and taurocholic acid were purchased from Shanghai Yuanye Biotechnology Co., Ltd.

### Animal handling

This study was carried out in accordance with the recommendations of guidelines of the experimental animal ethics committee of Jiangxi University of traditional Chinese Medicine. The protocol was approved by the experimental animal ethics committee of Jiangxi University of traditional Chinese Medicine. Specific pathogen free (SPF) Sprague–Dawley rats (female, 180–220 g) were obtained from the Laboratory Animal Center of Wuhan University (Wuhan, China). Prior to the experiment, all rats were acclimated for 1 week under standard laboratory conditions. Subsequently, 40 rats were randomly divided into cervicitis model and control groups. In the rat model, cervicitis was induced by phenol mucilage according to a previously described method (Ma et al., 2013; Song et al., 2017). Control group rats were treated identically with physiological saline.

### Histopathological analysis of cervicitis model rats

Cervical tissue samples were collected from rats on day 7 after establishing the cervicitis model. The tissues were processed and embedded in paraffin blocks. Sections (Thickness, 5 μm) sections were prepared and mounted on slides, deparaffinized in xylene, and dehydrated in alcohol before staining with hematoxylin and eosin (HE). Histopathological analysis of the tissues from control and cervicitis model rats was performed by examination under Olympus CX31 microscope (Olympus Corporation, Japan).

### Rat plasma sample collection and preparation

Eye orbital venous blood samples were collected into eppendorf tube (EP tube) which is coated with heparin sodium from the rats on day 7 after establishing the cervicitis model. Plasma was obtained after 20 min by centrifugation at 4,500 r/min for 10 min at 4°C. All plasma samples were stored at −80°C prior to sample preparation.

A working IS solution of 2-chloro-L-phenylalanine (5.31 μg/mL) was prepared in methanol. Plasma samples (50 μL) were added to 200 μL of the working IS solution. A total of 780 μL rat plasma sample (20 μL of each rat plasma sample, one from model group died in the experimental progress) were added to 3,120 μL of the working IS solution to generate a quality control (QC) sample for validating the reproducibility of the method and UPLC-QTOF-MS/MS stability. Pretreated samples were vortexed for 3 min, and then centrifuged (15,000 r/min, 4°C) for 10 min. The supernatant was transferred into a sample bottle and stored at 4°C for MS analysis of cervicitis rat metabolomics.

### Human plasma sample collection and preparation

This study was carried out in accordance with the recommendations of guidelines of the Ethics Committee of the Affiliated Hospital of Jiangxi Institute of Traditional Chinese Medicine, with written informed consent from all subjects. All subjects gave written informed consent in accordance with the Declaration of Helsinki. The protocol was approved by the Ethics Committee of the Affiliated Hospital of Jiangxi Institute of Traditional Chinese Medicine. Plasma sample collection of patients was conducted in Affiliated Hospital of Jiangxi Institute of Traditional Chinese Medicine from March 1, 2016 to February 28, 2017. A total of 193 patients with the following inclusion and exclusion criteria were enrolled. *Inclusion criteria*: (a), Age of 20–40 years old, weight of 45–70 kg; (b), No medication was taken in a month and normal diet; (c), Consistent with the diagnostic criteria for mycoplasma cervicitis (Workowski and Bolan, 2015); (d), Participate in the investigation voluntarily and is able to cooperate with the examination. *Exclusion criteria*: (e), Merge with venereal diseases such as gonorrhea, syphilis, trichomonas vaginitis, urethritis, etc.; (f), Combine with other local or systemic Inflammatory diseases which are irrelative to this study including pharyngitis, bronchitis, gastritis, etc. (g), Menstrual, gestational, and lactating women. It turns out patients in control group (20 patients, aged of 31.15 ± 5.78 years old, weight of 55.45 ± 6.34 kg) meet with the requirements of inclusion and exclusion criteria except the consistent with the diagnostic criteria for mycoplasma cervicitis. And patients in mycoplasma cervicitis group (20 patients, aged of 29.70 ± 5.05 years old, weight of 53.60 ± 6.65 kg) comply with the requirements of inclusion and exclusion criteria. Human blood was collected into EP tube which is coated with heparin sodium. All human plasma samples were obtained after 20 min by centrifugation at 4,500 r/min for 10 min at 4°C and stored at −80°C prior to sample preparation.

Human plasma sample (50 μL) were added to 200 μL of the working IS solution. 20 μL of each human plasma sample were added to 3,200 μL of the working IS solution to obtain a QC sample. Pretreated samples were vortexed for 3 min, and then centrifuged (15,000 r/min, 4°C) for 10 min. The supernatant was transferred into a sample bottle and stored at 4°C for MS analysis of human cervicitis metabolomics.

### LC-MS conditions

The UPLC analysis was carried out on an ACQUITY H-CLASS instrument (Waters Corp., Milford, MA, USA) equipped with an automatic degasser, a quaternary pump, and an autosampler. An ACQUITY UPLC™ HSS T3 column (100 × 2.1 mm, 1.7 μm; Waters Corp.) was applied for chromatographic separation. The mobile phases consisted of 0.1% formic acid/water (A) and acetonitrile (B). The mobile phase gradient was as follows: 0–3 min, 5–20% B; 3–5 min, 20–40% B; 5–9 min, 40–60% B; 9–16 min, 60–65% B; 16–18 min, 65–80% B; 18–21 min, 80–95% B; 21–23 min, 95–5% B; and 23–25 min, 5% B. The flow rate was set to 0.35 mL/min, with an injection volume set to 5 μL, and the column oven set at 30°C.

MS/MS detection was conducted on a Triple TOF™ 5,600+ system (equipped with a Duo Spray source) for ions in both positive and negative modes with high resolution (AB SCIEX, Foster City, CA, USA). In the positive mode, the electrospray ionization was applied with the following parameters: ion spray voltage, 4,500 V; ion source temperature, 500°C; curtain gas, 25 psi; nebulizer gas (GS 1), 50 psi; heater gas (GS 2), 50 psi; and declustering potential (DP), 80 V. In the information dependent acquisition (IDA) experiment, the collision energy (CE) was set at 35 eV, and the collision energy spread (CES) was (±) 10 eV. In the negative mode, the electrospray ionization was applied with the following parameters: ion spray voltage, −4,500 V; ion source temperature, 500°C; curtain gas, 25 psi; GS 1, 50 psi; GS 2, 50 psi; and DP, −100 V. In the IDA, CE was set at −30 eV, CES was (±) 10 eV. In both the positive and negative ion modes, the mass ranges were set at m/z 50–1,250 Da for TOF-MS scans and TOF MS/MS scans. The MS/MS fragmentation was selected from the eight most intense ions for each TOF-MS scan. Dynamic background subtraction (DBS) was applied to match the IDA tests for UPLC-QTOF-MS/MS.

### Validation of the analytical protocol

Prior to the plasma analysis, the precision of the instrument and the method repeatability were validated by duplicate analysis of six injections QC samples prepared as described previously. To investigate plasma stability, the QC sample was detected at 0, 6, 12, 18, 24, and 48 h, after preparation. In addition, QC samples were analyzed every 10 injections during plasma sample analysis in both the positive and negative modes. The retention time and intensity of each peak were determined using PeakView software 1.2.0 with XIC manager (AB SCIEX) and statistical analysis of relative standard deviations (RSD) was performed to validate the analytical method (Table S1).

### Data processing and statistical analysis

All the plasma samples were analyzed by UPLC-QTOF-MS/MS and the raw data were processed by MarkerView v1.2.1 software (AB SCIEX). The data processing involved retention time correction and sample normalization using the IS 2-chloro-L-phenylalanine. The processed data were exported from MarkerView v1.2.1 software, and then imported into the SIMCA-P 14.1 (Umetrics, Sweden) to perform multivariate statistical data analysis. All the data was scaled using the pareto scaling algorithm and autofitted for multivariate analysis. First, unsupervised principal component analysis (PCA) was performed to create an overview, and a DModX was applied to remove outliers. Supervised orthogonal partial least squares-discriminant analysis (OPLS-DA) was then applied to distinguish the contribution of the detected variables to the discrimination between the groups (Li et al., 2017). The established model was assessed by calculating the *R*^2^ and *Q*^2^ values. A large *R*^2^ (close to 1) is a desirable condition for a good model. *Q*^2^ indicates how well the model predicts new data based on cross-validation and a large *Q*^2^ (*Q*^2^ > 0.5) indicates good predictability. A large *Q*^2^ (*Q*^2^ > 0.5) indicates good predictability. Large *R*^2^ and *Q*^2^ values are not sufficient for a good model; therefore, the permutation test was applied to assess the predictability of the model.

Following screening with two-tailed independent Student's *t*-tests (*P* < 0.05) by SPSS 19.0 Statistics (IBM) and Variable Importance in Projection (obtained from OPLS-DA, VIP > 1) (Lu et al., 2016), the filtered metabolites were identified as potential biomarkers using the following online databases: HMDB (http://www.hmdb.ca/), METLIN (https://isometlin.scripps.edu/), Mass Bank (http://www.massbank.jp/), Chemspider (http://www.chemspider.com/), combining the MS/MS spectrometry of standard and PeakView software 1.2.0 with XIC manager. Pathway analysis was then performed with MetaboAnalyst (http://www.metaboanalyst.ca/) and the Kyoto Encyclopedia of Genes and Genomes (KEGG; http://www.kegg.jp/).

## Results

### Histological examination

**Figure 1:**
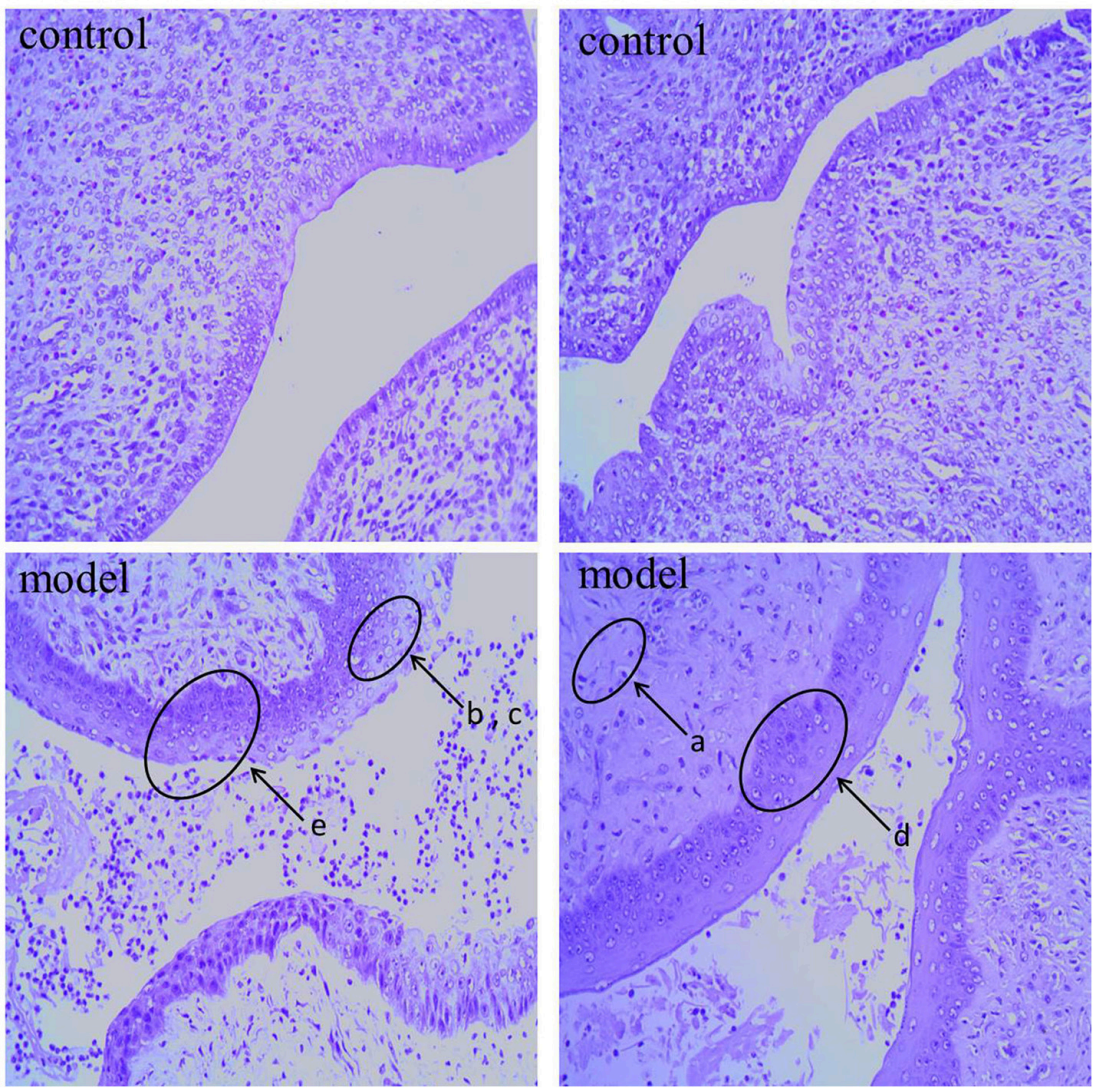
Representative H&E stain of cervix sections from control group rat and cervicitis group rat on 7th day: the lowercase letter a-e means pathological features in model group.

As shown in Figure 1, the histological features were obtained by hematoxylin and eosin (HE) staining of cervical tissue from control and cervicitis model rats. In the model group, we observed inflammatory cell infiltration (a), epithelial cell degeneration (b), necrosis(c), glandular destruction, squamous epithelial layer incrassation (d), and some visible epithelial erosion (e). In contrast, these pathological abnormalities were rare in the control group by comparison.

### Validation of analytical method

Prior to plasma sample detection, the QC samples of rat and human were detected to validate the reproducibility of the method and the stability of UPLC-QTOF-MS/MS system, respectively. During the detection, the QC samples were inserted between every 10 experimental samples to monitor the batches. The plasma samples of rat and human were detected separately. The total ion chromatograms (TICs) of QC sample injections are shown in the supplementary material (see Figure S1 online). Visual inspections suggested almost complete overlap between the positive and negative chromatograms. Further examination was conducted by comparison of the ion intensities and retention time deviation of a selection of eight common extracted ion chromatograms. Eight typical peaks in positive ion mode and eight typical peaks in negative ion mode were used. This method was validated by referring to previous literature in non-targeted metabolomics (Gu et al., 2015; Sui et al., 2017). In both the positive and negative ion modes, the relative standard deviations (RSD) of ion intensities and retention time deviation were below 10 and 0.14%, respectively. Detailed information is provided in Figure S1 and Table S1.

### Metabolic profile of rat samples

#### Multivariate analysis of rat metabolic profile

**Figure 2:**
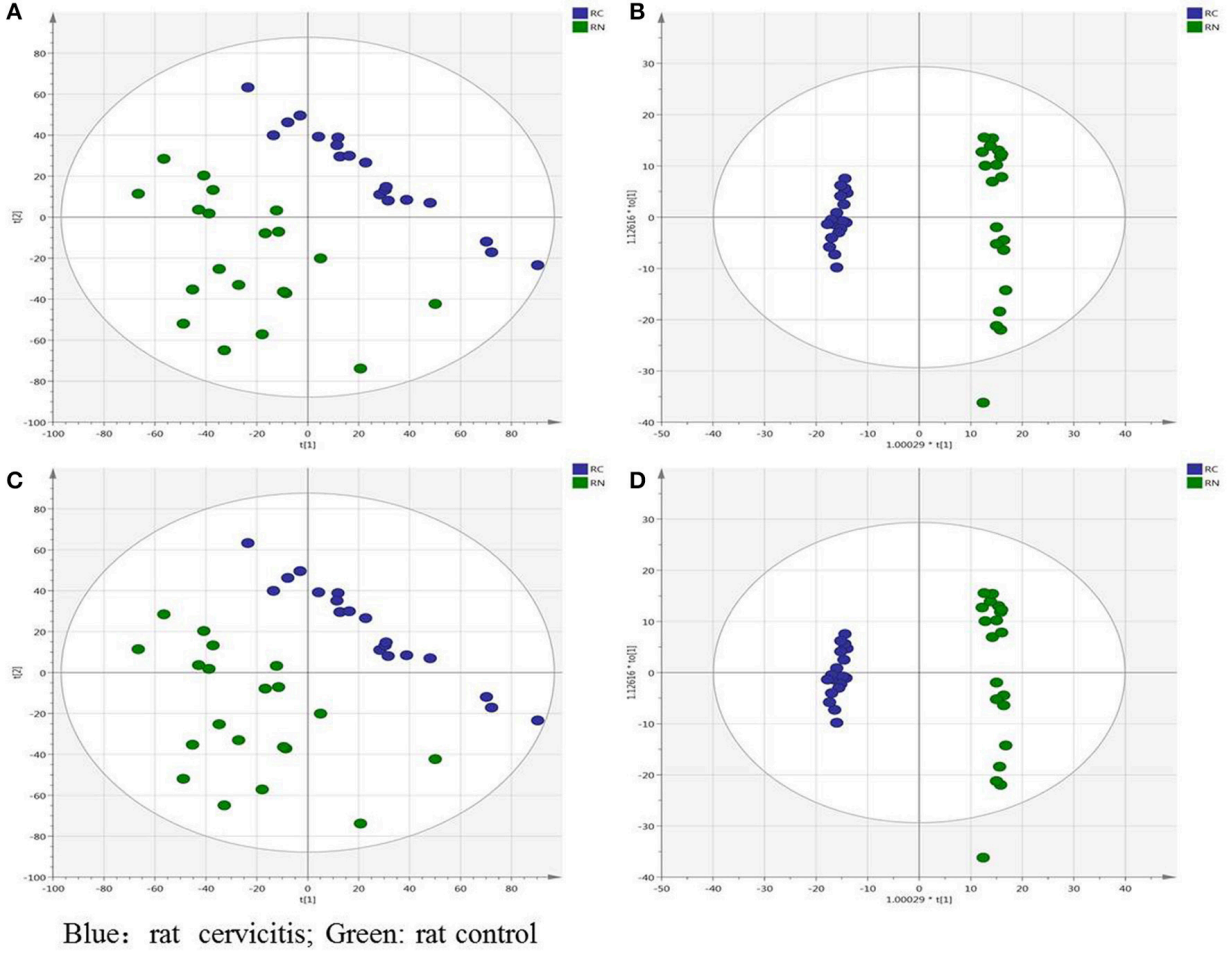
Overview of the rat plasma samples: **(A)** PCA score plot in positive ion mode; **(B)** OPLS-DA score plot in positive ion mode; **(C)** PCA score plot in negative ion mode; **(D)** OPLS-DA score plot in negative ion mode (green is rat control group, blue is cervicitis rat group).

Figures S2A,B show representative TICs of rat plasma samples in positive and negative ion modes, respectively. Multivariate analysis was performed on SIMCA-P 14.1 with the processed data exported from MarkerView v1.2.1 software. To investigate the intra differences between different groups, the principal component analysis (PCA) and orthogonal projections to latent structures discriminant analysis (OPLS-DA) analysis were utilized to search the differentiating variables. The PCA score plot showed that the plasma datasets of different groups were clearly separated both in the positive (Figure 2A) and negative ion modes (Figure 2C). After excluding outliers based on the DModX plot (it turns out no outliers in PCA analysis), the OPLS-DA analysis was performed to distinguish the differences between the control and model groups (Figures 2B,D). The datasets were divided into two clusters clearly identified in both the positive and negative ion modes. Furthermore, the relevant R^2^Y and Q^2^Y values were applied to assess the quality of the OPLS-DA models. For rat plasma metabolic profiles, R^2^Y and Q^2^Y were 0.989 and 0.957, respectively, in the positive ion mode and 0.981 and 0.959, respectively, in the negative ion mode. These values indicated that the presence of few irrelevant model terms and superior predictability parameters of the OPLS-DA models. To eliminate over-fitting effects, random effects, and to evaluate the predictive ability, the permutation of 200 tests was performed. Generally, lower *Q*^2^ values to the left compared to the original points (on the right) and intersection of the vertical axis (on the left) by the regression line of the *Q*^2^ points at, or below zero, strongly indicates the validity of the original model. *R*^2^ values to the left that are lower than the original point to the right are also an indication of the validity of the original model The parameters obtained in this study indicated that the OPLS-DA models were reliable with good predictability (see Figures S3A,B).

#### Identification of rat cervicitis potential biomarkers

**Figure 3:**
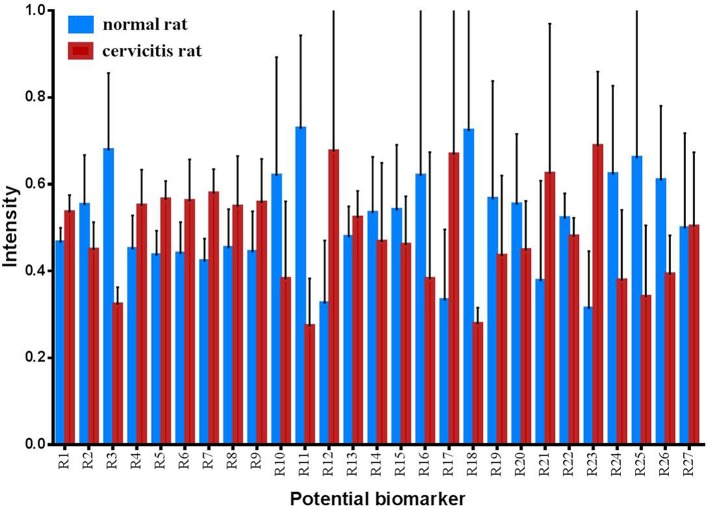
Changes for rat potential biomarkers between control group and cervicitis group (RN, control group; RC, cervicitis group).

**Figure 4:**
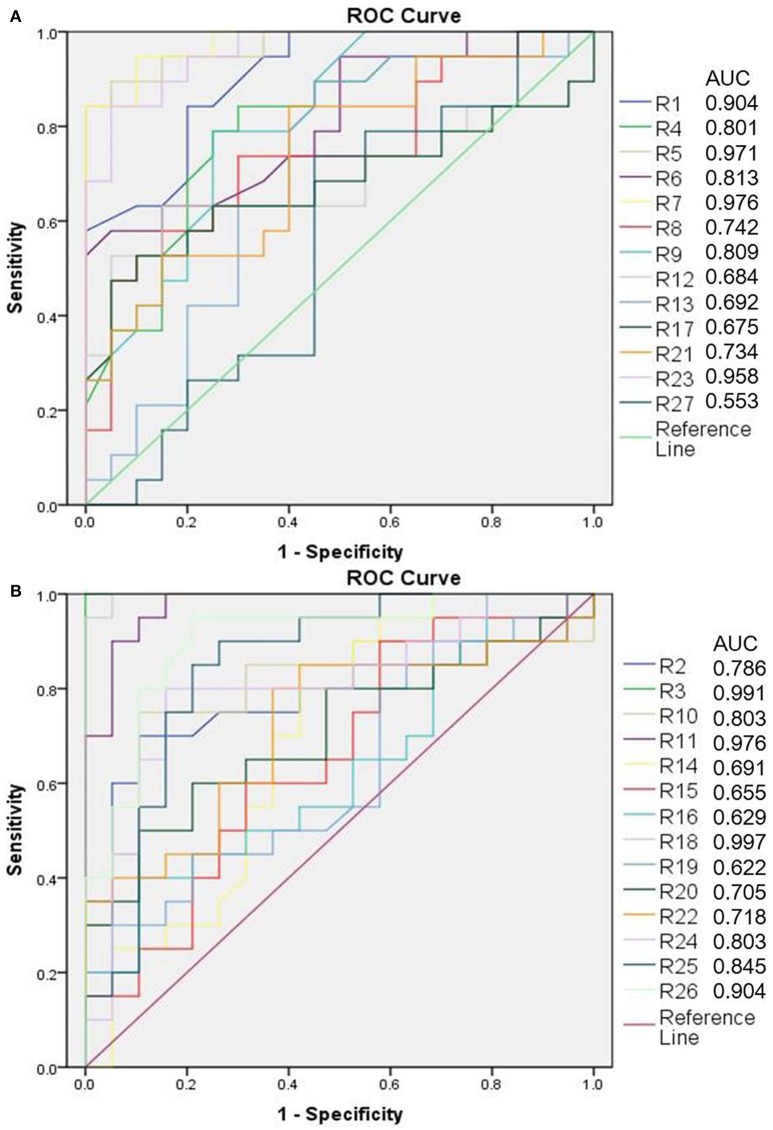
ROC analysis of rat potential biomarkers (**A** is up-regulated metabolites, **B** is down-regulated metabolites).

The OPLS-DA model was built to distinguish the metabolites that contributed to discrimination of the rat sample profile. Differential metabolites (VIP > 1) were then screened as contributive variables for apparent discrimination. Those filtered metabolites were subjected to independent *t*-test using SPSS 19.0. The metabolites which adhered to the parameters of VIP > 1 and *P* < 0.05 were identified as candidate metabolites using the online databases. The accurate mass determination of their precursor ions and MS/MS fragments were conducted by using the high resolution UPLC-QTOF-MS/MS. The accurate mass and its MS/MS fragments were then matched with standards (Figure S4) and those metabolites provided by online database such as HMDB (http://www.hmdb.ca/), METLIN (https://isometlin.scripps.edu/), Mass Bank (http://www.massbank.jp/), Chemspider (http://www.chemspider.com/). The error between extraction mass value and experimental mass value was less than 5 ppm. The candidate metabolites which matched with those requirements would be regarded as potential biomarkers. In total, 27 metabolites were identified as potential biomarkers by comparisons of the rat metabolomics profiles of the cervicitis model and control groups (Table 1 and Figure 3). Furthermore, to evaluate the significance of each potential biomarker, receiver operating characteristic curve (ROC) analysis was generated (Figure 4). According to area under curve (AUC) (Table 1), 3-methyl-5-propyl-2-furanundecanoic acid, etiocholanolone, LysoPC(18:3), LysoPC(20:5), stearoylcarnitine, 9,10-epoxyoctadecenoic acid, PI(20:4/0:0), thymidine, LysoPC(18:2), LysoPC(20:4), PC(O-18:1/2:0), SM(d18:0/16:1), S-(PGA2)-glutathione, taurocholic acid were identified as particularly significant biomarkers in the rat cervicitis metabolomics profile.

**Table 1:** Identification of potential biomarkers of rat plasma samples between control group and cervicitis module group.

**No**.	**Name**	**Retention time**	**M.F**	**Error (ppm)**	**Ion mode**	**VIP**	***P*-value**	**AUC**
1	3-Methyl-5-propyl-2-furanundecanoic acid	18.63	C_19_H_32_O_3_	−1.1	[M+H]+	1.02	1.19E-06	0.904
2	Acetylcarnitine^#^	1.11	C_9_H_17_NO_4_	−2.6	[M+H]+	1.15	1.49E-03	0.786
3	Etiocholanolone^#^	16.71	C_19_H_30_O_2_	−0.5	[M+NH_4_]+	2.87	3.29E-10	0.991
4	LysoPC(18:2)	10.48	C_26_H_50_NO_7_P	−3.1	[M+H]+	10.22	4.41E-04	0.801
5	LysoPC(18:3)^#^	11.25	C_26_H_48_NO_7_P	−4.2	[M+H]+	2.48	1.71E-09	0.971
6	LysoPC(20:4)	10.55	C_28_H_50_NO_7_P	−4.7	[M+H]+	10.75	8.20E-05	0.813
7	LysoPC(20:5)	10.47	C_28_H_48_NO_7_P	−3.6	[M+H]+	2.14	8.73E-11	0.976
8	LysoPC(22:6)	10.51	C_30_H_50_NO_7_P	−4.4	[M+H]+	4.22	7.30E-03	0.742
9	PC(O-18:1/2:0)	15.77	C_28_H_56_NO_7_P	−3.5	[M+H]+	1.32	8.81E-04	0.809
10	SM(d18:0/16:1)	20.19	C_39_H_79_N_2_O_6_P	−1.2	[M+H]+	7.74	2.92E-03	0.803
11	Stearoylcarnitine	15.11	C_25_H_49_NO_4_	−1.9	[M+H]+	1.21	7.00E-10	0.976
12	Tetracosahexaenoic acid	9.96	C_24_H_36_O_2_	−2.6	[M+H]+	1.95	1.35E-02	0.684
13	Tryptophan	2.7	C_11_H_12_N_2_O_2_	−1.7	[M+H]+	1.25	4.48E-02	0.692
14	Pregnenolone	9.44	C_21_H_32_O_2_	−2.8	[M+H]+	2.34	9.94E-03	0.691
15	Tetradecanoylcarnitine	9.7	C_21_H_41_NO_4_	−3.1	[M+H]+	1.08	3.40E-02	0.655
16	20-Hydroxyeicosatetraenoic acid	13.18	C_20_H_32_O_3_	−0.4	[M-H]-	1.45	4.55E-02	0.629
17	3b,7a-Dihydroxy-5b-cholanoic acid	9.93	C_24_H_40_O_4_	−0.9	[M-H]–	2.01	1.85E-02	0.675
18	9,10-Epoxyoctadecenoic acid	11.95	C_18_H_32_O_3_	−3.4	[M-H]–	3.86	3.55E-04	0.997
19	Arachidonic acid	8.38	C_20_H_32_O_2_	−1.4	[M-H]–	1.35	4.47E-02	0.622
20	Eicosapentaenoic acid	18.62	C_20_H_30_O_2_	−2.1	[M-H]–	1.70	2.55E-02	0.705
21	Glycocholic acid	6.74	C_26_H_43_NO_6_	−1.8	[M-H]–	1.26	1.24E-02	0.734
22	LysoPE(0:0/20:0)	14.72	C_25_H_52_NO_7_P	−1.6	[M-H]–	1.55	1.47E-02	0.718
23	PI(20:4/0:0)	11.12	C_29_H_49_O_12_P	−1	[M-H]–	7.49	3.93E-09	0.958
24	S-(PGA2)-glutathione	14.63	C_30_H_47_N_3_O_10_S	0.3	[M-H]–	1.15	2.10E-04	0.803
25	Taurocholic acid	6.1	C_26_H_45_NO_7_S	−1.8	[M-H]–	2.68	2.20E-03	0.845
26	Thymidine	1.83	C_10_H_14_N_2_O_5_	−0.3	[M-H]–	1.40	1.96E-05	0.904
27	3-Carboxy-4-methyl-5-propyl-2-furanpropionic acid	5.81	C_12_H_16_O_5_	−3.6	[M-H]–	2.24	3.55E-03	0.553

p-value is t-test comparing normal rat and cervicitis rat; confidence intervals of AUC is 95%;

# *means the biomarker was identified in both rat model and human disease*.

#### Analysis of metabolic pathways

**Figure 5:**
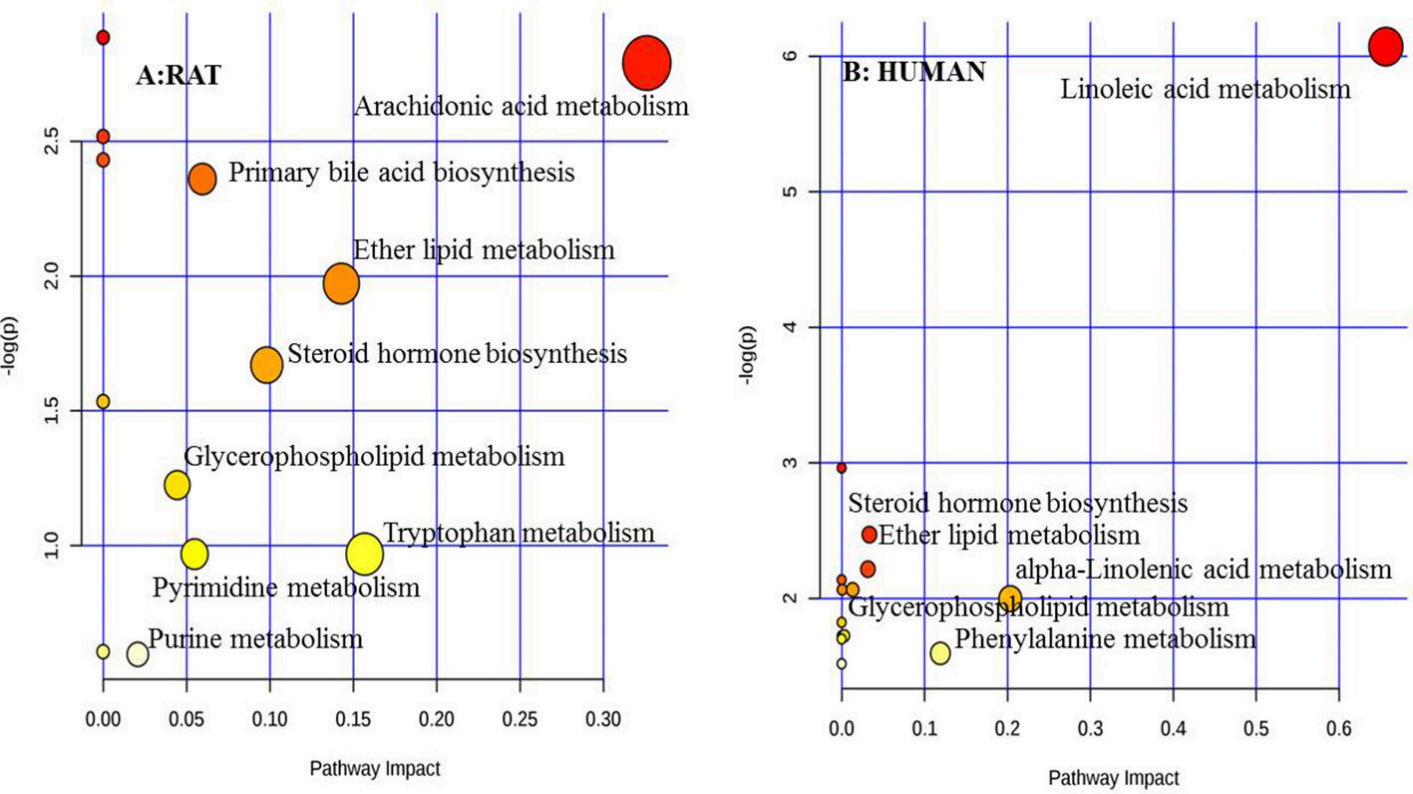
Overview of metabolic pathway analysis: **(A)** for rat plasma metabolic, **(B)** for human individual plasma metabolic. Pathway analysis is performed using MetaboAnalyst 3.0 (http://www.metaboanalyst.ca).

All the 27 potential biomarkers were subjected to perform metabolic pathway analysis (MetPA) using the Kyoto Encyclopedia of Genes and Genomes (KEGG) online database and MetaboAnalyst 3.0 (Xia and Wishart, 2010). An overview of the pathway analysis shown in Figure 5A reflected the metabolic network of cervicitis. For the rat plasma samples, metabolic pathways were identified as arachidonic acid metabolism, primary bile acid biosynthesis, ether lipid metabolism, steroid hormone biosynthesis, glycerophospholipid metabolism, tryptophan metabolism, pyrimidine metabolism, and purine metabolism.

### Metabolic profile of human samples

#### Multivariate analysis of human metabolic profile

**Figure 6:**
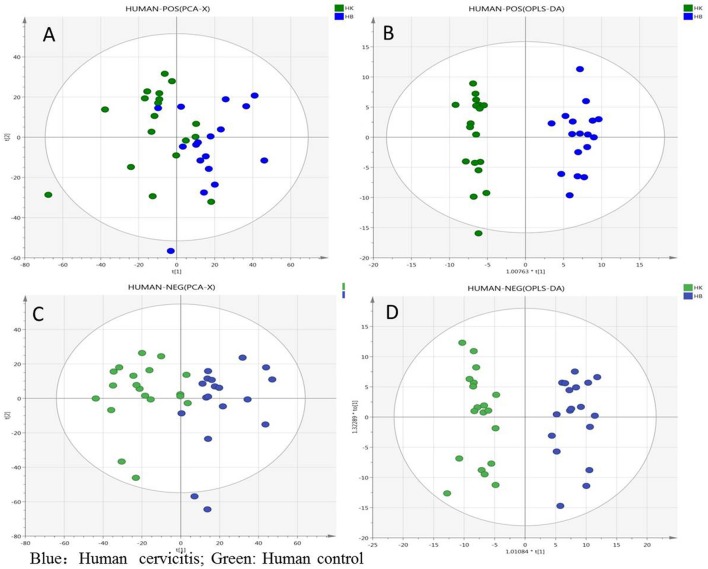
Overview of the human plasma samples: **(A)** PCA score plot in positive ion mode; **(B)** OPLS-DA score plot in positive ion mode; **(C)** PCA score plot in negative ion mode; **(D)** OPLS-DA score plot in negative ion mode (green is human normal group, blue is cervicitis patients group).

As shown in Figure S2 online, the representative TICs of human plasma samples were generally similar to those of rat plasma samples. Multivariate analysis was performed using SIMCA-P 14.1 with the processed data exported from MarkerView v1.2.1 and the PCA and OPLS-DA analysis were performed to identify the differentiating variables between plasma samples of healthy women and cervicitis patients. According to PCA score plots, the plasma samples of the different groups were clearly separated in the positive and negative ion modes. OPLS-DA analysis was then performed to determine differentiating variables after eliminating outlying samples. As shown in Figure 6, the plasma samples were clearly divided into two clusters in both the positive and negative ion modes, although data obtained in the negative ion mode showed better separation than those in the positive ion mode. R^2^Y and Q^2^Y values were also calculated to assess the quality of the human plasma OPLS-DA model. The R^2^Y and Q^2^Y values were 0.966 and 0.849, respectively, in the positive ion mode, and 0.929 and 0.763, respectively, in the negative ion mode. In both modes, the difference between the R^2^Y and Q^2^Y values were less than 0.2, indicating few irrelevant model terms or outlying data-points. Furthermore, the permutation test indicated that the validity of the original model (see Figures S3C,D). Therefore, the parameters obtained in this study indicated that the OPLS-DA models of human cervicitis were reliable with good predictability.

#### Identification of potential biomarkers of human cervicitis

**Figure 7:**
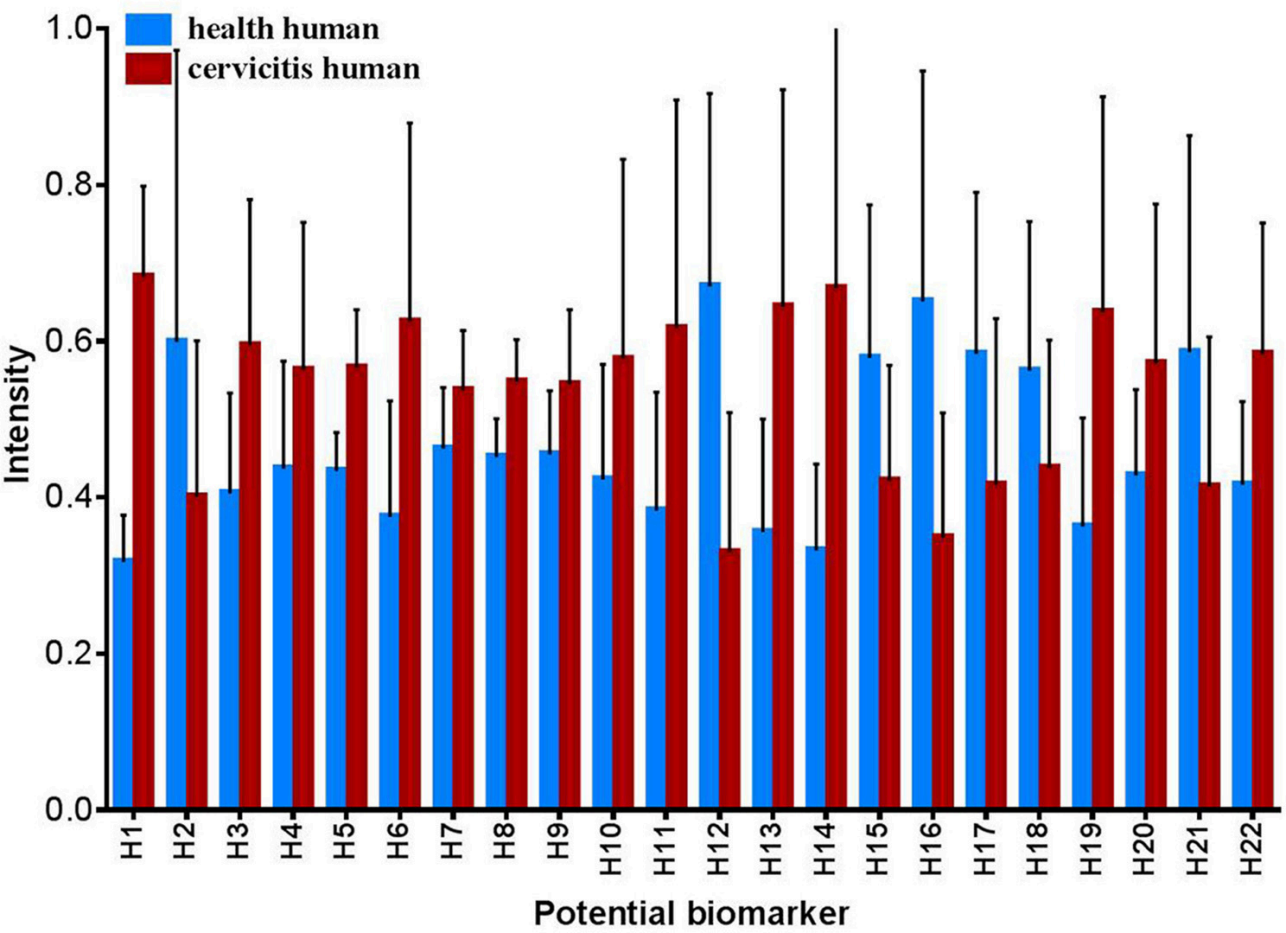
Changes for human potential biomarkers between health group and cervicitis group (HN, health human group, HC, cervicitis human group).

**Figure 8:**
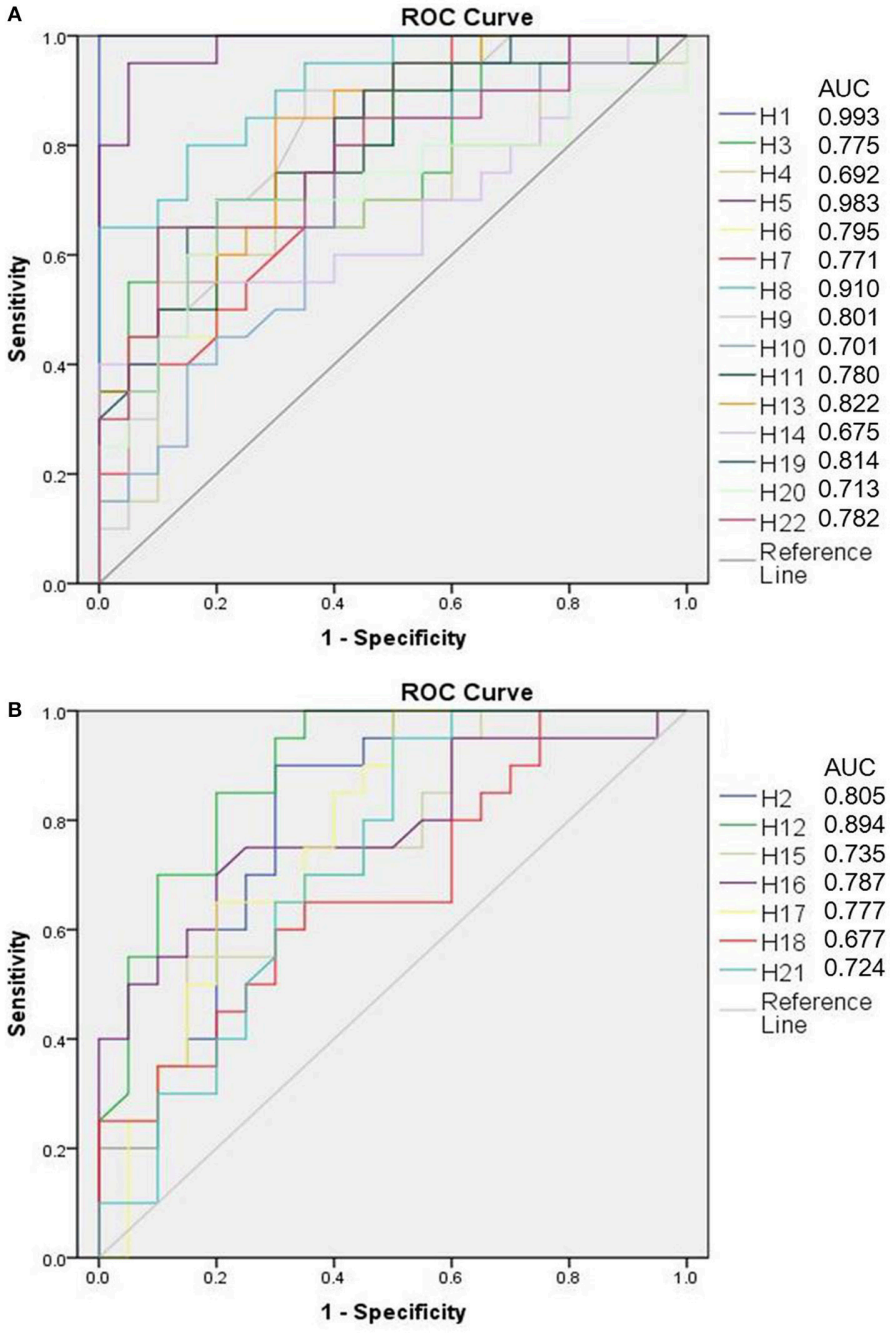
ROC analysis of human potential biomarkers (**A** is up-regulated metabolites, **B** is down-regulated metabolites).

Based on OPLS-DA models for both the positive and negative ion modes, significant differentially expressed metabolites were identified in the human sample profile. In total, 22 metabolites that adhered to the parameters of VIP > 1 and *P* < 0.05 were identified as potential biomarkers of human cervicitis (Table 2 and Figure 7). Furthermore, to evaluate the significance of each potential biomarker, ROC analysis was generated (Figure 8). According to AUC values (Table 2), 1-Acetoxy-2-hydroxy-16-heptadecyn-4-one, Etiocholanolone, L-Valine, 9-Decenoylcarnitine, LysoPC(18:3), Phytosphingosine, Vaccenyl carnitine, LysoPE(18:1/0:0) were identified as the most important potential biomarkers of human cervicitis.

**Table 2:** Identification of potential biomarkers of human plasma samples between healthy women and cervicitis patients.

**No**.	**Name**	**Retention time**	**M.F**	**Error (ppm)**	**Ion mode**	**VIP**	***P*-value**	**AUC**
1	1-Acetoxy-2-hydroxy-16-heptadecyn-4-one	11.25	C_19_H_32_O_4_	0.3	[M+H]+	3.62	5.86E-13	0.993
2	9-Decenoylcarnitine	6.33	C_17_H_31_NO_4_	2.5	[M+H]+	1.99	1.27E-02	0.805
3	Acetylcarnitine^#^	0.9	C_9_H_17_NO_4_	0.9	[M+H]+	1.87	5.63E-04	0.775
4	Cortisol	5.92	C_21_H_30_O_5_	1.5	[M+H]+	1.11	3.41E-02	0.692
5	Etiocholanolone^#^	16.9	C_19_H_30_O_2_	1	[M+NH_4_]+	2.05	5.55E-08	0.983
6	Linoelaidyl carnitine	10.44	C_25_H_45_NO_4_	2.1	[M+H]+	2.27	9.78E-04	0.795
7	L-Phenylalanine	1.9	C_9_H_11_NO_2_	−0.2	[M+H]+	1.01	8.81E-03	0.771
8	L-Valine	0.78	C_5_H_11_NO_2_	0.2	[M+H]+	1.48	1.96E-06	0.910
9	LysoPC(18:3)	11.34	C_26_H_48_NO_7_P	−0.9	[M+H]+	1.16	3.73E-03	0.801
10	LysoPC(P-18:0)	12.83	C_26_H_54_NO_6_P	−0.3	[M+H]+	1.25	4.04E-02	0.701
11	PC(O-18:1/2:0)	15.99	C_28_H_56_NO_7_P	−0.5	[M+H]+	1.61	6.38E-03	0.780
12	Phytosphingosine	7.46	C_18_H_39_NO_3_	3.5	[M+H]+	3.36	6.16E-05	0.894
13	Vaccenyl carnitine	11.83	C_25_H_47_NO_4_	0.8	[M+H]+	2.44	6.37E-04	0.822
14	12,13-Dihydroxy-9-octadecenoic acid	19.24	C_18_H_34_O_4_	1.2	[M-H]–	1.46	1.61E-02	0.675
15	9-Hydroxy-10,12-octadecadienoic acid	12	C_18_H_32_O_3_	2.6	[M-H]–	1.58	7.37E-03	0.735
16	3-Oxoandrostan-17-yl hydrogen sulfate	7.12	C_19_H_30_O_5_S	1.9	[M-H]–	2.28	3.98E-04	0.787
17	gamma-Linolenic acid	18.79	C_18_H_30_O_2_	−1.3	[M-H]–	1.66	2.01E-02	0.777
18	Linoleic acid	20.17	C_18_H_32_O_2_	0.1	[M-H]–	1.27	3.71E-02	0.677
19	LysoPE(18:1/0:0)	11.93	C_23_H_46_NO_7_P	0	[M-H]–	2.08	6.06E-04	0.814
20	LysoPE(18:2/0:0)	10.39	C_23_H_44_NO_7_P	3.4	[M-H]–	1.12	1.25E-02	0.713
21	Palmitoleic acid	19.51	C_16_H_30_O_2_	1.2	[M-H]–	1.60	3.66E-02	0.724
22	PI(20:4/0:0)^#^	11.31	C_29_H_49_O_12_P	−0.7	[M-H]–	3.68	3.32E-07	0.782

p-value is t-test comparing normal women and cervicitis patients; confidence intervals of AUC is 95%;

# *means the biomarker was identified in both rat model and human disease*.

#### Analysis of metabolic pathways

The 22 identified potential biomarkers were imported into MetaboAnalyst 3.0 for pathway analysis. For the human plasma samples, metabolic pathways were identified as linoleic acid metabolism, steroid hormone biosynthesis, ether lipid metabolism, alpha-linolenic acid metabolism, glycerophospholipid metabolism, and phenylalanine metabolism. An overview of the pathway analysis is shown in Figure 5B.

## Discussion

In current, diagnostic tests for *M. genitalium* are not available (Workowski and Bolan, 2015; Gaydos, 2017). Due to this deficiency, *M. genitalium* infected cervicitis can hardly be perceived in clinical. Therefore, the study was mainly focused on patients infected with mycoplasma cervicitis. When the body infected with disease, endogenous metabolites in plasma will be affected, accordingly. As a local inflammation, cervicitis will also influence the level of endogenous metabolites in plasma to some extent. In addition, plasma sample were widely used for discovery of potential biomarkers for the diagnosis of diseases such as polycystic ovary syndrome, cervical cancer, chronic low-grade inflammation, etc (Sun et al., 2012; Hasim et al., 2013; Garbett et al., 2014; Pietzner et al., 2017; Yang et al., 2017). As a result, a comprehensive understanding of the pathogenesis of cervicitis is urgently required. In this study, a plasma metabolomic profiling strategy was adopted to clarify the pathogenesis of cervicitis and identify potential biomarkers in humans as well as a rat model of cervicitis induced by phenol mucilage (Gao and Wu, 1997; Gao, 1999; Qu et al., 2008; Zhou et al., 2010). This model is reliable and effectively manipulated and more importantly, cervicitis can be induced under experimental conditions without influencing other tissues. This metabolomic profiling revealed obvious differences between healthy and cervicitis model rats. Further studies of human cervicitis revealed significant differences between the plasma metabolomics profiles of cervicitis patients and healthy women.

**Figure 9:**
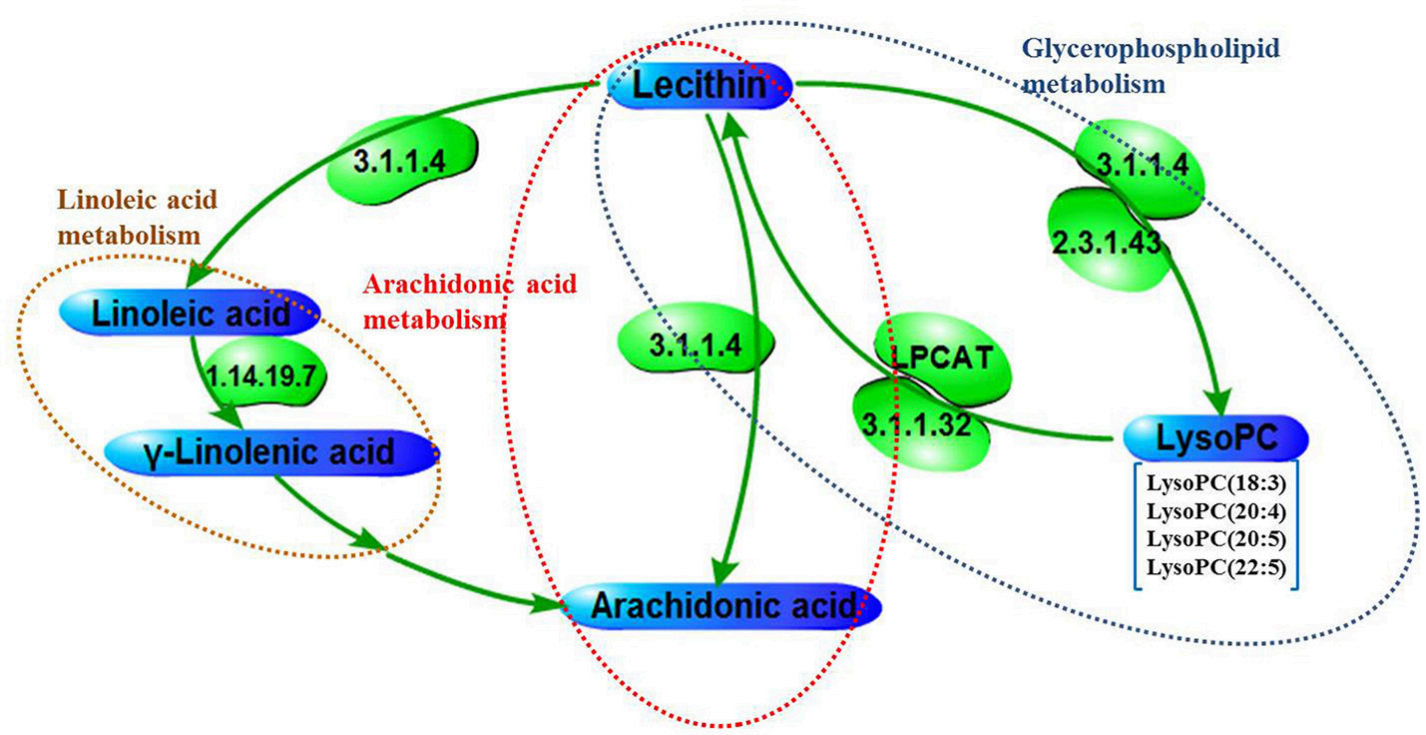
The metabolic pathway networks of potential biomarkers in response to cervicitis, The word in green means enzyme according to KEGG (http://www.genome.jp/kegg/).

The results has displayed that there were some overlaps between the metabolic analysis of the human and rat cervicitis, namely, glycerophospholipid metabolism, ether lipid metabolism, steroid hormone biosynthesis. In addition, arachidonic acid metabolism and linoleic acid metabolism were interacted closely. Based on the metabolomics profiling and pathway analysis, a metabolite pathway map was constructed (Figure 9). Tables 1, 2 have showed there were also some overlaps for biomarkers between rat and human cervicitis, such as lysophosphatidylcholines [LysoPC (18:2), LysoPC (18:3), LysoPC (20:4), LysoPC 20:5), LysoPC (22:6)], polyunsaturated fatty acid(linoleic acid, arachidonic acid, 20-hydroxyeicosatetraenoic acid), carnitine derivatives (9-decenoylcarnitine, acetylcarnitine, vaccenyl carnitine, linoelaidyl carnitine, tetradecanoylcarnitine, stearoylcarnitine), PC(O-18:1/2:0) and PI(20:4/0:0). These similarities could be helpful to study human cervicitis pathology and therapeutic mechanism by using a rat cervicitis model.

As it is shown, there are some differences between metabolite pathways of the human and rat cervicitis to some extent. The metabolomics profiling has clarified that rat cervicitis refers to primary bile acid biosynthesis, tryptophan metabolism, pyrimidine metabolism, and purine metabolism. On the other hand, human cervicitis was revealed to be related with alpha-linolenic acid metabolism, and phenylalanine metabolism. This study also revealed some potential biomarkers in rat cervicitis or human cervicitis such as tryptophan, glycocholic acid, L-Valine, L-Phenylalanine, etc.

Glycerophospholipid metabolism, which is widely found in many species, was identified as an important pathway in both of human and rat cervicitis in this study. Our results revealed the involvement of a series of lysophosphatidylcholines (LysoPCs), including LysoPC (18:2), LysoPC (18:3), LysoPC (20:4), LysoPC 20:5), LysoPC (22:6). Lysophosphatidylcholine is a monoglycerophospholipid in which a phosphorylcholine moiety occupies a glycerol substitution site and is generated by the degradation of glycerophosphocholines by phospholipase A(2)[PLA(2)]. There are significant amounts of lysophosphatidylcholine in plasma and several studies have indicated that it may be a proinflammatory mediator, causing inflammatory responses through disrupting endothelial barrier function (Huang et al., 2005; Du et al., 2015; Inose et al., 2015; Tanaka et al., 2016). Previous reports have also revealed increased lysophosphatidylcholine levels in some inflammatory diseases and gynecological disorders (Xiao et al., 2000; Yoder et al., 2014; Yang et al., 2017). As shown in Tables 1, 2, the level of LysoPC in cervicitis patients was higher than those in the control group. These indicate that LysoPC may be a potential biomarker of cervicitis, and glycerophospholipid metabolism may be closely associated with occurrence of cervicitis.

Both the human and rat cervicitis were involved with the metabolic pathway of ether lipid metabolism. As is shown in Tables 1, 2, PC(O-18:1/2:0) is the potential biomarker of both human and rat cervicitis. It is also named with 2-Acetyl-1-(9Z-octadecenyl)-sn-glycero-3-phosphocholine by International Union of Pure and Applied Chemistry (IUPAC). PC(O-18:1/2:0) is an intermediate in ether lipid metabolism and an ether lipid with platelet-activating factor (PAF) functions which has an acetyl group instead of an acyl chain at the second position (SN-2). PAF is produced by macrophages during inflammation and infections, and it could initiate an inflammatory response in allergic reactions (Chiang et al., 2006; Belayev et al., 2008; McIntyre et al., 2015; Borges et al., 2017). The level of PAF may indicate the occurrence of inflammation. In our study, the levels of PC(O-18:1/2:0) were significantly higher(*P* < 0.01) in human and rat cervicitis group compared to control group, respectively. Therefore, it may prefigure that cervicitis may closely involve the ether lipid metabolism and potential biomarker of PC(O-18:1/2:0).

Both the human and rat cervicitis were involved with the metabolic pathway of steroid hormone biosynthesis. It is acutely regulated by pituitary trophic hormones and other steroidogenic stimuli (Stocco, 2001). The metabolites of pregnenolone and etiocholanolone play an important role in steroid hormone biosynthesis. It could induce immunostimulation and leukocytosis. According to Tables 1, 2, etiocholanolone is the potential biomarker of both human and rat cervicitis. The AUC values of etiocholanolone were 0.991 and 0.983 for rat cervicitis and human cervicitis, respectively. The AUC values indicate that the etiocholanolone may be a significant biomarker for cervicitis.

As shown in Figures 7, 8, PI (20:4/0:0) were identified as potential biomarkers both in human cervicitis and rat cervicitis. PI (20:4/0:0) is a subclass of glycerophosphoinositols, which is a lipid comprising a common glycerophosphate skeleton linked to at least one fatty acyl chain and an inositol moiety. But the biofunction of PI (20:4/0:0) remains unknown. However, ROC analysis (Figures 7, 8) indicate that PI (20:4/0:0) may play an important role in discriminating the metabolomics profiles of these two groups, which implicates this molecule may be a potential biomarker of cervicitis.

As a form of gynecological inflammation, the pathogenesis of cervicitis is related to arachidonic acid (AA) metabolism. The polyunsaturated, essential fatty acid AA and its metabolites play a central role in regulating inflammatory signaling pathways (Ma et al., 2016). AA is the substrate for the synthesis of biologically active compounds, such as leukotrienes, prostaglandins, thromboxanes, which act as regulators of inflammatory cytokine production and immune function (Jayaraja et al., 2016; Monk et al., 2016). In addition, linoleic acid metabolism was identified as a metabolic pathway linked to cervicitis (Figure 9). In our study, we identified gamma-linolenic acid and linolenic acid as potential biomarkers involved in this metabolic pathway. Furthermore, linolenic acid, a polyunsaturated omega-6 fatty acid, is important in the biosynthesis of AA and therefore, some prostaglandins, leukotrienes (LTA_4_, LTB_4_, LTC_4_), and thromboxane (TXA_2_). Previous study showed that arachidonic acid and linoleic acid were mianly poly-unsaturated fatty acids in the plasma membrane which could produce oxidized pro-inflammatory lipid intermediates with the conversion by lipoxygenases catalyze (Samala et al., 2017). In our study, the level of these poly-unsaturated fatty acids in control group is higher than that in disease group. Thus, our metabolomics profiling of rat and human cervicitis indicate that linoleic acid metabolism and AA metabolism may be associated with the development of this disorder.

Sometimes, it would be extremely difficult to comprehensively understand the clinical pathogenesis of the disease. The progress of sample collection and selection is also time-consuming and arduous. And it is especially complex to research the mechanism of action of pro-drugs or active constituents in holism. Without any targets, it could be even more difficult. Therefore, researching a clinical disease by building a corresponding animal model can put through an abecedarian exploration and thus provide a reliable foundation for subsequent clinical study. In this study, the metabolic pathway of rat cervicitis model, to some extent, is in accordance with those of human cervicitis, which could provide a novel perspective for searching effective mechanism for cervicitis of drugs or active constituents in clinical.

## Conclusion

In this study, plasma metabolomics profiling of both cervicitis patients and a rat model of the disease was successfully established based on UPLC-QTOF-MS/MS as an appropriate technique for the identification of potential biomarkers of cervicitis. Furthermore, the metabolomics profile of the rat model of cervicitis provided some important information for the investigation of human cervicitis. Our results suggest that the metabolic pathways involved in cervicitis include AA, ether lipid, glycerophospholipid, and linoleic acid metabolism. In addition, AA, linoleic acid, lysophosphatidylcholine, PI (20:4/0:0) were implicated as potential biomarkers of cervicitis for use in clinical practice. In addition, this study showed the rat model was successfully created and applied to understand the pathogenesis of cervicitis. However, the role of these biomarkers in the pathogenesis of cervicitis requires confirmation in further proteomics and transcriptomics studies.

## Author contributions

XZ wrote this main manuscript text and performed the data analysis. The animal experiment including histopathological analysis was conducted by XZ and JL, together. And the human plasma sample was collected by BX and BW. In addition, SL gave the contribution to plasma sample detection. As for data process, it is conducted by YY and MH. HO contributed significantly to analysis and manuscript preparation. WX designed the work that led to the submission, acquired data, and played an important role in interpreting the results. YF revised the manuscript and approved the final version. And SY contributed to the conception of the study.

### Conflict of interest statement

The authors declare that the research was conducted in the absence of any commercial or financial relationships that could be construed as a potential conflict of interest. The reviewer GP and handling Editor declared their shared affiliation.

## Supplementary material

Figure S1 Total ion chromatogram (TIC) of 16 QC sample injections: **(A)** for positive ion mode; **(B)** for negative ion mode (P1-P8 represents the 8 peaks in positive ion mode, N1-N8 represents the 8 peaks in negative ion mode).

Figure S2 Total ion chromatogram (TIC): **(A)** Rat plasma sample in positive ion mode; **(B)** Rat plasma sample in negative ion mode; **(C)** Human plasma sample in positive ion mode; **(D)** Human plasma sample in negative ion mode.

Figure S3 **(A)** Permutations test plot for OPLS-DA (200 tests) in positive ion mode for rat plasma samples; **(B)** Permutations test plot for OPLS-DA (200 tests) in negative ion mode for rat plasma samples; **(C)** Permutations test plot for OPLS-DA (200 tests) in positive ion mode for human plasma samples; **(D)** Permutations test plot for OPLS-DA (200 tests) in negative ion mode for human plasma samples.

Figure S4 MS/MS spectrometry of standard and metabolite.

Table S1 Retention time and intensity of eight typical peaks extracted from 16 QC sample injections.
