# Supplementary figures and images for: Comparative Metabolomics Analysis of Cervicitis in Human Patients and a Phenol Mucilage-Induced Rat Model Using Liquid Chromatography Tandem Mass Spectrometry
Source: Front Pharmacol. 2018 Apr 4;9:282. doi: 10.3389/fphar.2018.00282 (PMC5893906; doi:10.3389/fphar.2018.00282)

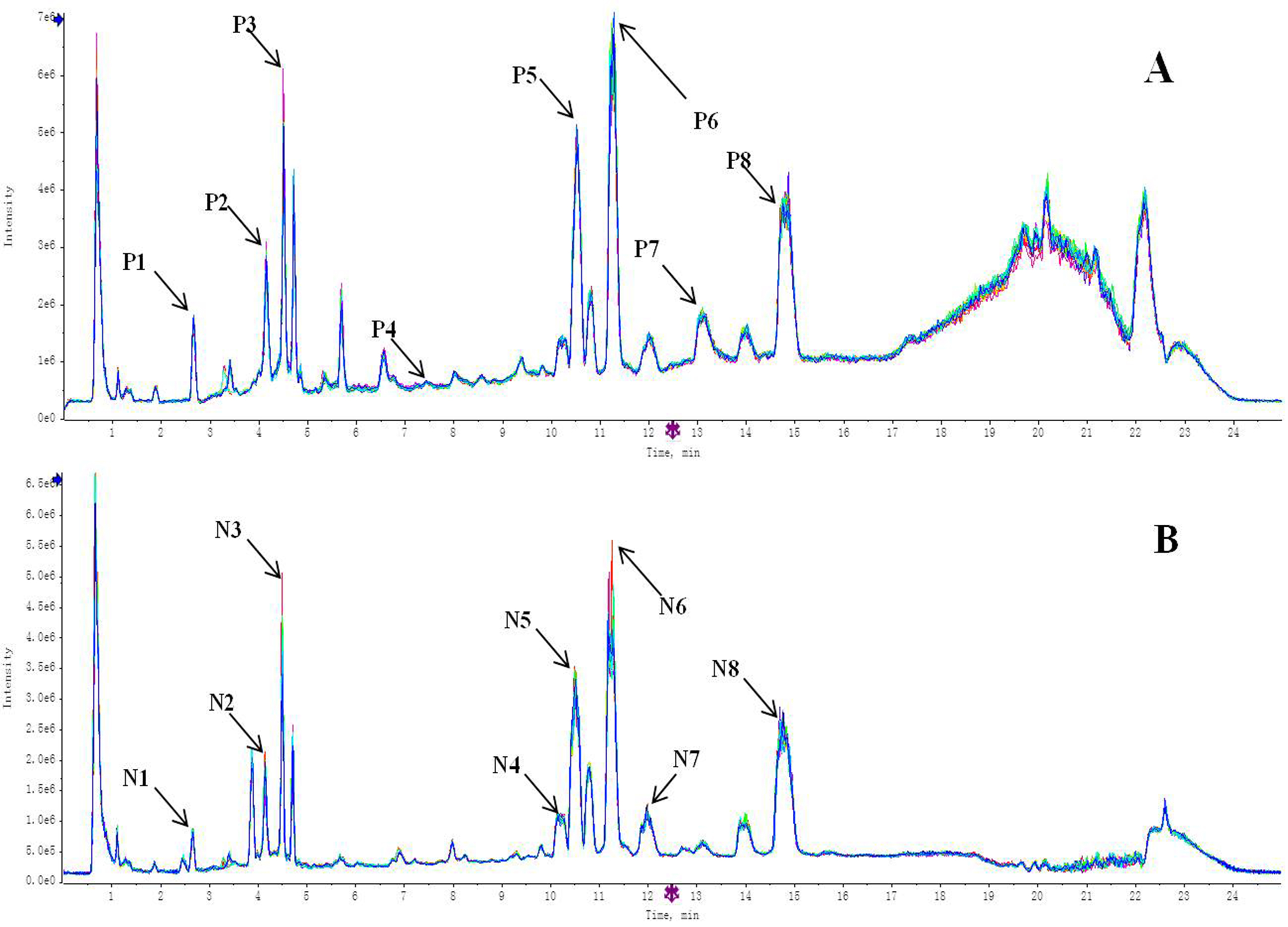

Supplement: Figure S1 — Total ion chromatogram (TIC) of 16 QC sample injections: (A) for positive ion mode; (B) for negative ion mode (P1-P8 represents the 8 peaks in positive ion mode, N1-N8 represents the 8 peaks in negative ion mode). [file Image1.JPEG]

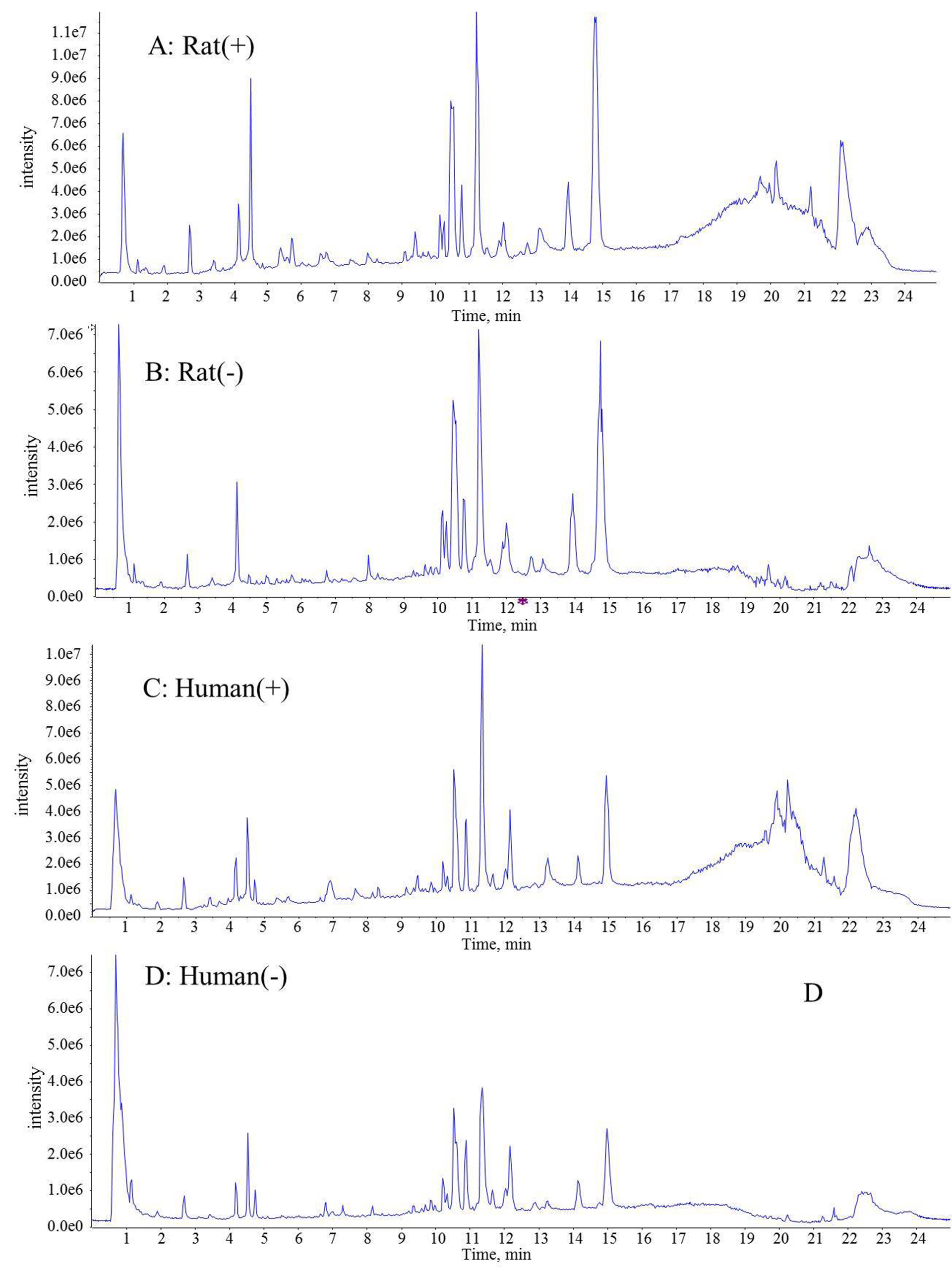

Supplement: Figure S2 — Total ion chromatogram (TIC): (A) Rat plasma sample in positive ion mode; (B) Rat plasma sample in negative ion mode; (C) Human plasma sample in positive ion mode; (D) Human plasma sample in negative ion mode. [file Image2.JPEG]

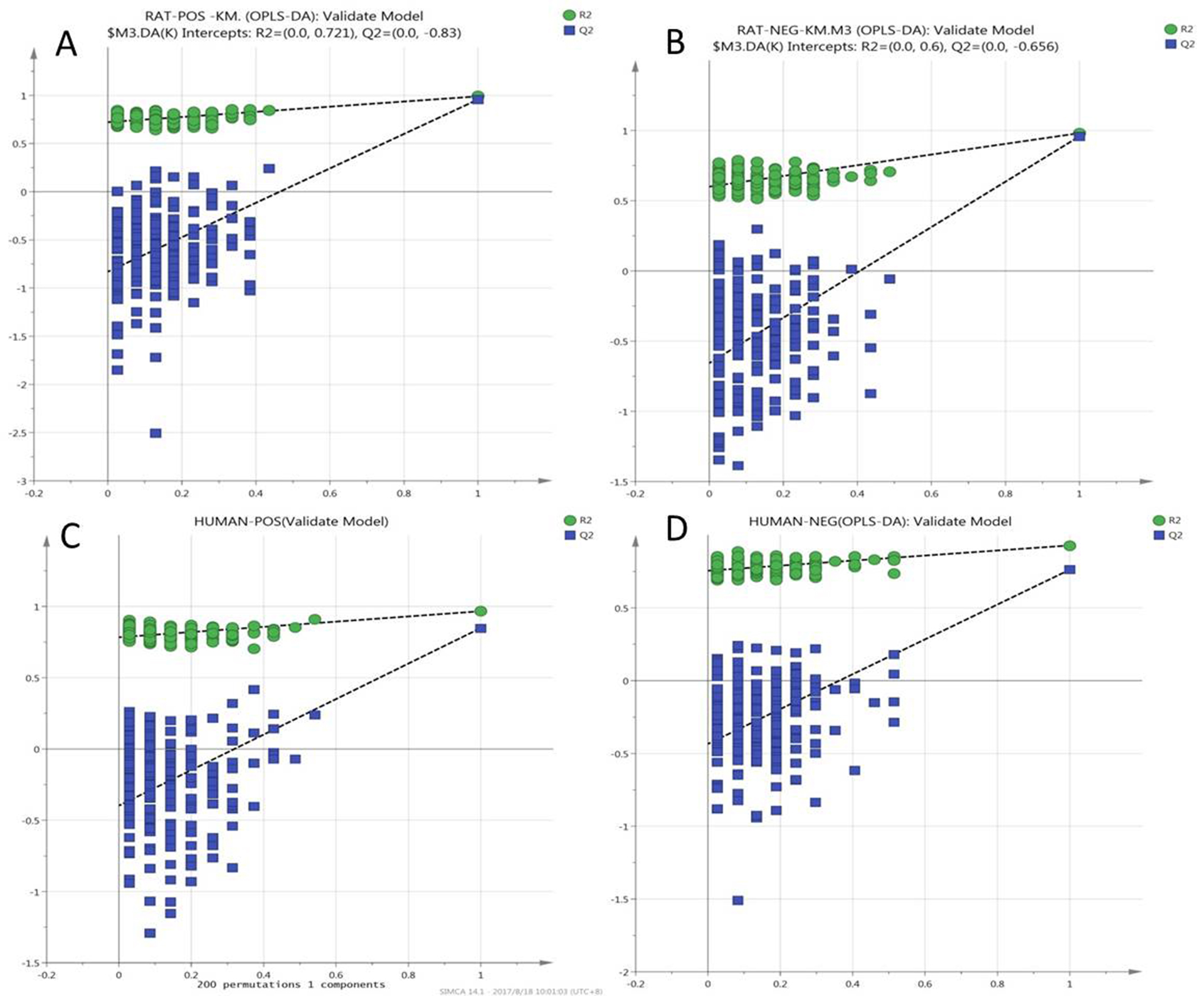

Supplement: Figure S3 — (A) Permutations test plot for OPLS-DA (200 tests) in positive ion mode for rat plasma samples; (B) Permutations test plot for OPLS-DA (200 tests) in negative ion mode for rat plasma samples; (C) Permutations test plot for OPLS-DA (200 tests) in positive ion mode for human plasma samples; (D) Permutations test plot for OPLS-DA (200 tests) in negative ion mode for human plasma samples. [file Image3.JPEG]
