# Supplementary material for: Comparative Metabolomics Analysis of Cervicitis in Human Patients and a Phenol Mucilage-Induced Rat Model Using Liquid Chromatography Tandem Mass Spectrometry
Source: Front Pharmacol. 2018 Apr 4;9:282. doi: 10.3389/fphar.2018.00282 (PMC5893906; doi:10.3389/fphar.2018.00282)

Supplementary Information

Supplementary Figure 4: MS/MS spectrometry of standards and metabolites.

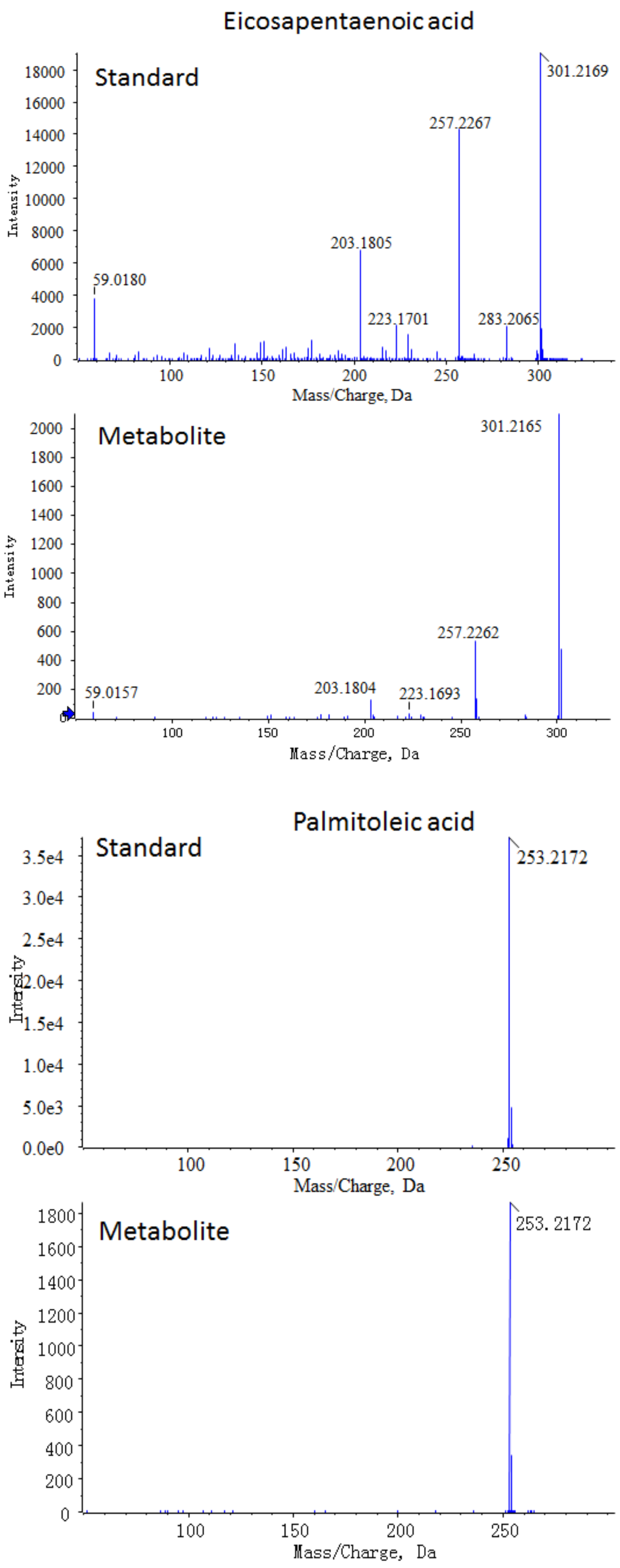

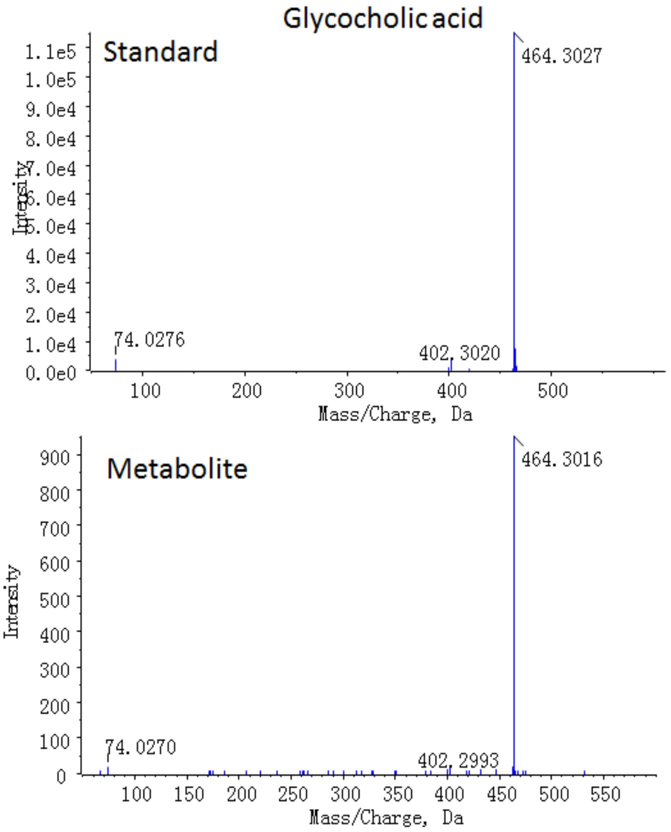
Glycocholic acid
Standard
464.3027
74.0276
402.3020
Intensity
Mass/Charge, Da
Metabolite
464.3016
74.0270
402.2993
Intensity
Mass/Charge, Da

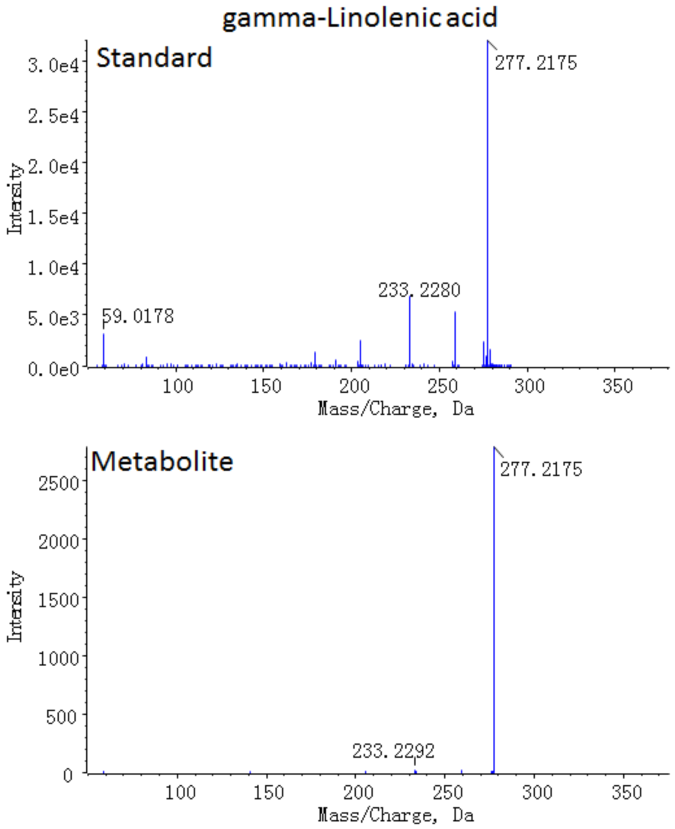
gamma-Linolenic acid
Standard
277.2175
233.2280
59.0178
Intensity
Mass/Charge, Da
Metabolite
277.2175
233.2292
Intensity
Mass/Charge, Da

Arachidonic acid

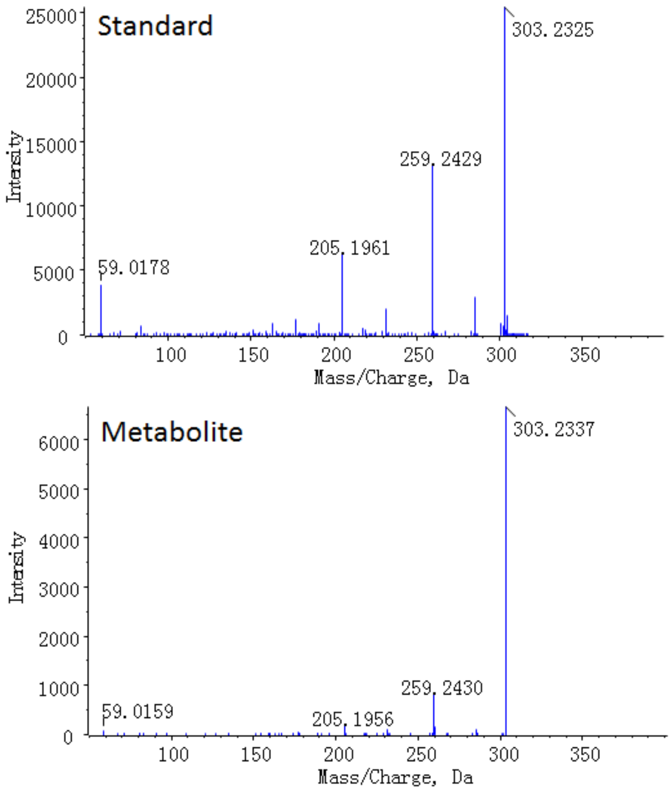

Thymidine

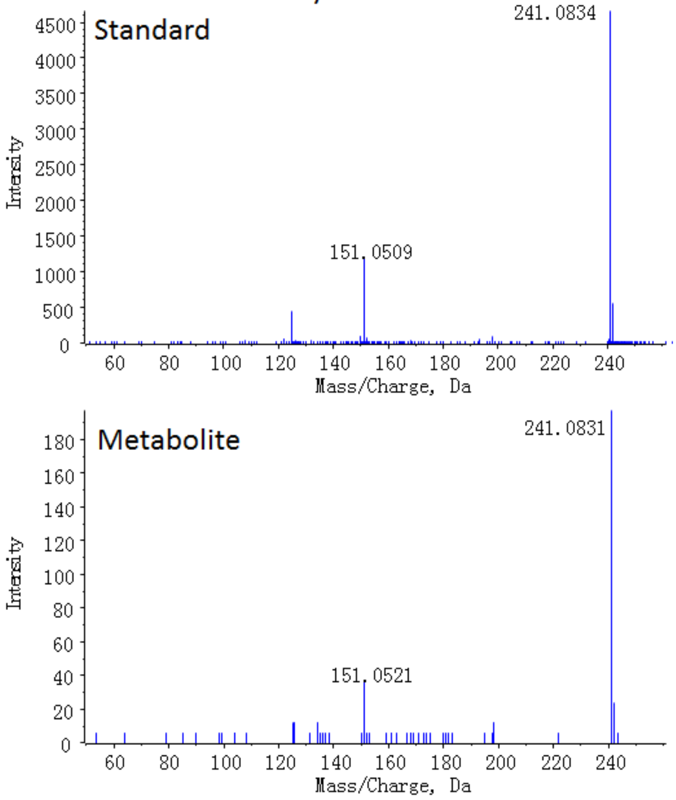

Linoleic acid

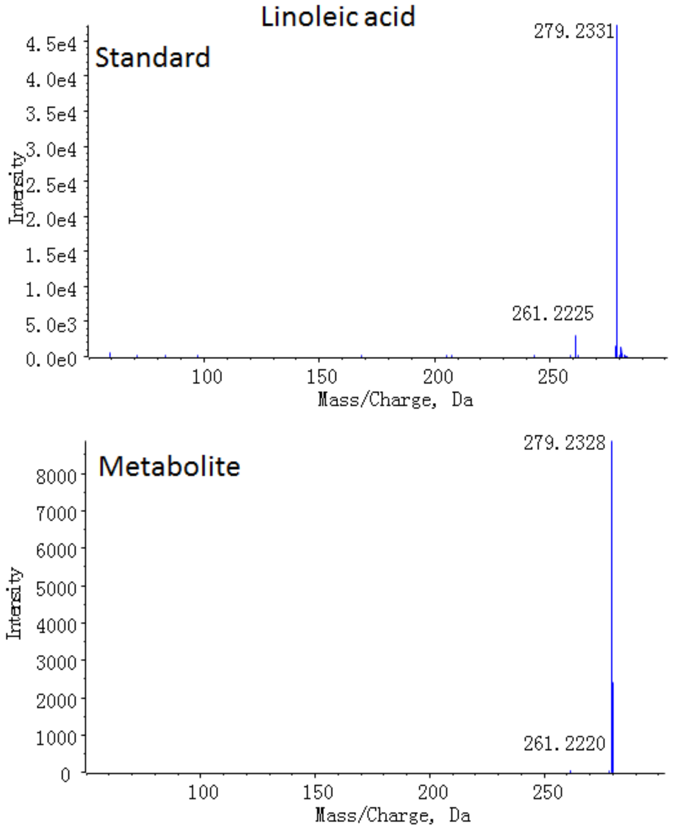

Taurocholic acid

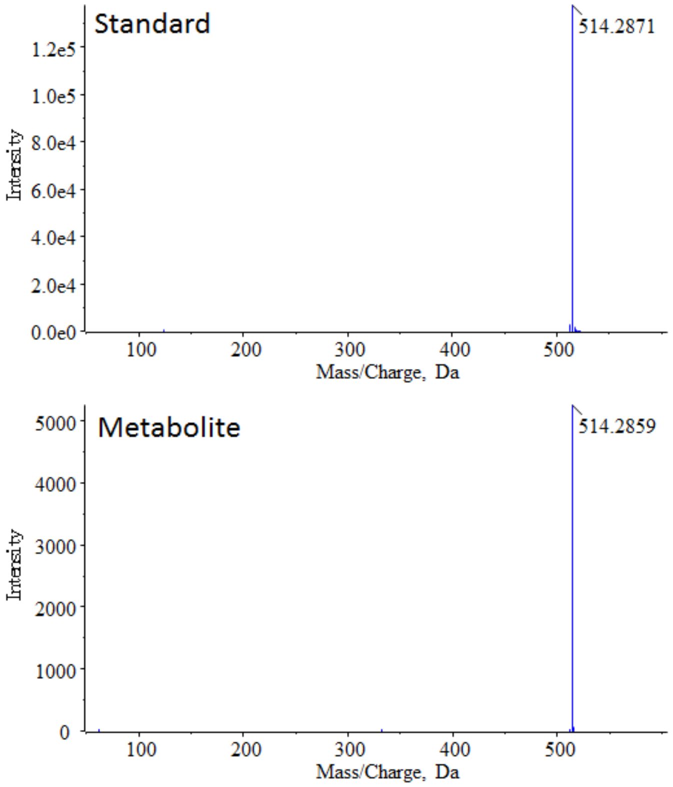

L-Phenylalanine

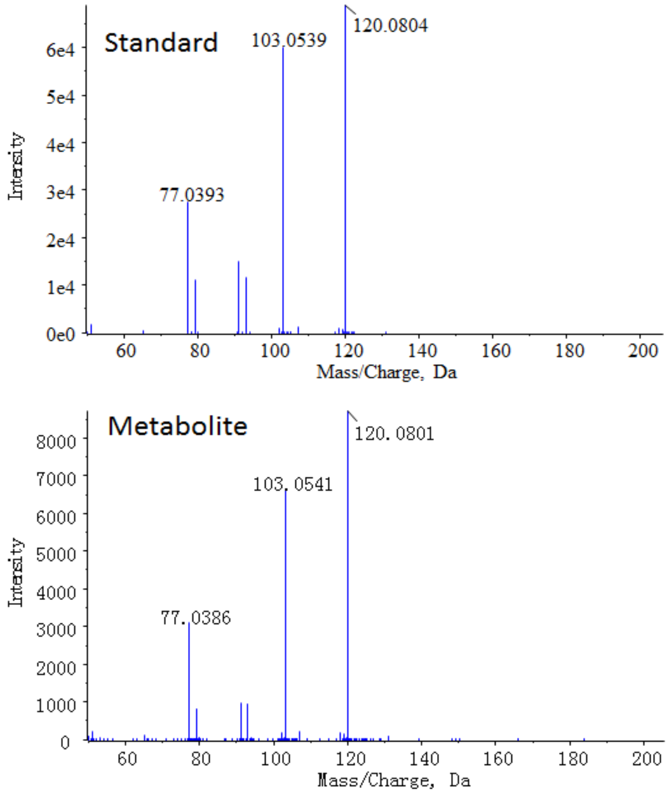

L-Tryptophan

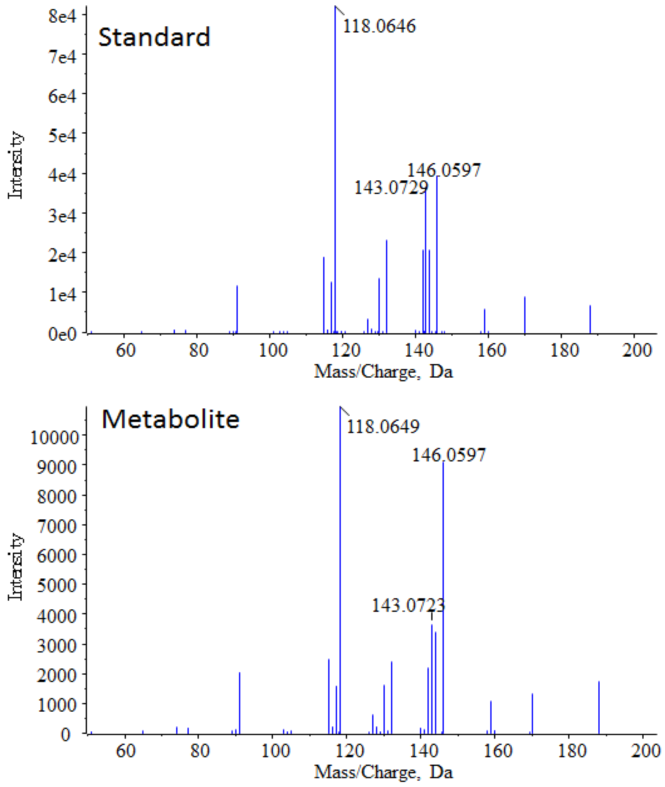

Supplement: Figure S4 — MS/MS spectrometry of standard and metabolite. [file Image4.PDF]
