# Supplementary material for: Comparative Metabolomics Analysis of Cervicitis in Human Patients and a Phenol Mucilage-Induced Rat Model Using Liquid Chromatography Tandem Mass Spectrometry
Source: Front Pharmacol. 2018 Apr 4;9:282. doi: 10.3389/fphar.2018.00282 (PMC5893906; doi:10.3389/fphar.2018.00282)
Supplement: Table S1 — Retention time and intensity of eight typical peaks extracted from 16 QC sample injections. [file Table1.DOCX]

**Supplementary Information**

**Supplementary Table S1.** Retention time and intensity of eight typical peaks extracted from sixteen QC sample injections.

| **Peak name** | **MS** | **Ion mode** | **RSD** | |
| --- | --- | --- | --- | --- |
|  |  |  | **Intensity** | **Retention time** |
| P1 | 200.0473 | positive | 4.46% | 0.12% |
| P2 | 453.3445 | positive | 8.77% | 0.12% |
| P3 | 566.4266 | positive | 8.79% | 0.05% |
| P4 | 274.2743 | positive | 6.24% | 0.05% |
| P5 | 544.3393 | positive | 4.91% | 0.02% |
| P6 | 496.3412 | positive | 7.17% | 0.03% |
| P7 | 577.1353 | positive | 5.97% | 0.08% |
| P8 | 524.3716 | positive | 2.95% | 0.03% |
| N1 | 198.0329 | negative | 8.28% | 0.22% |
| N2 | 497.3345 | negative | 5.58% | 0.14% |
| N3 | 610.4173 | negative | 9.77% | 0.11% |
| N4 | 564.3305 | negative | 3.26% | 0.04% |
| N5 | 588.3188 | negative | 3.43% | 0.05% |
| N6 | 540.3308 | negative | 3.09% | 0.02% |
| N7 | 566.3465 | negative | 5.61% | 0.05% |
| N8 | 568.363 | negative | 3.16% | 0.04% |
